# Supplementary material for: Cdc25‐Mediated Activation of the Small GTPase RasB Is Essential for Hyphal Fusion and Symbiotic Infection of Epichloë festucae
Source: Mol Plant Pathol. 2026 Jan 28;27(1):e70210. doi: 10.1111/mpp.70210 (PMC12851848; doi:10.1111/mpp.70210)
Supplement: Supplementary file 1 — Figure S1: Alignment of the deduced amino acid sequence of EfCdc25 with Cdc25 from fungal species. [file MPP-27-e70210-s001.pdf]

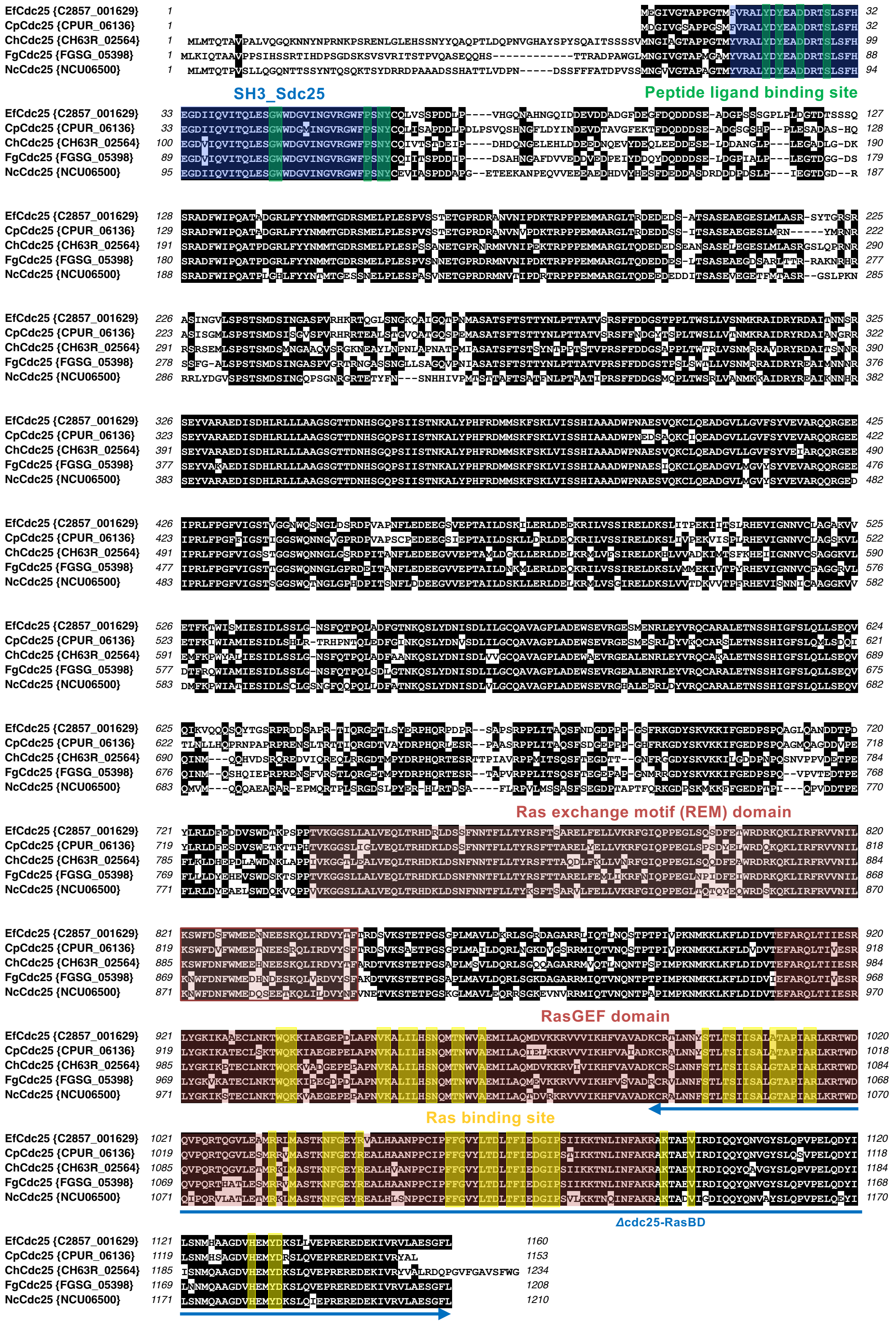

FIGURE S1 | Alignment of the deduced amino acid sequence of *Epichloë festucae* Cdc25 (EfCdc25; C2857_001629) with Cdc25 from *Claviceps purpurea* (CpCdc25; CPUR_06136), *Colletotrichum higginsianum* (ChCdc25; CH63R_02564), *Fusarium graminearum* (FgCdc25; FGSG_05398) and *Neurospora crassa* (NcCdc25; NUC06500). Src homology 3 (SH3) domain with a peptide ligand-binding site (SH3_Sdc25, blue), Ras exchange motif (REM) and Ras guanine nucleotide exchange factor (RasGEF) domains (pink) with conserved Ras-binding sites (yellow) are indicated. The region deleted in the *Δcdc25-RasBD* strains corresponds to the C-terminal portion of the RasGEF and Ras-binding domain is indicated by the horizontal blue double-headed arrow.
