# Supplementary material for: Cdc25‐Mediated Activation of the Small GTPase RasB Is Essential for Hyphal Fusion and Symbiotic Infection of Epichloë festucae
Source: Mol Plant Pathol. 2026 Jan 28;27(1):e70210. doi: 10.1111/mpp.70210 (PMC12851848; doi:10.1111/mpp.70210)
Supplement: Supplementary file 2 — Figure S2: Characterisation of E. festucae cdc25 mutant complemented strains. [file MPP-27-e70210-s002.pdf]

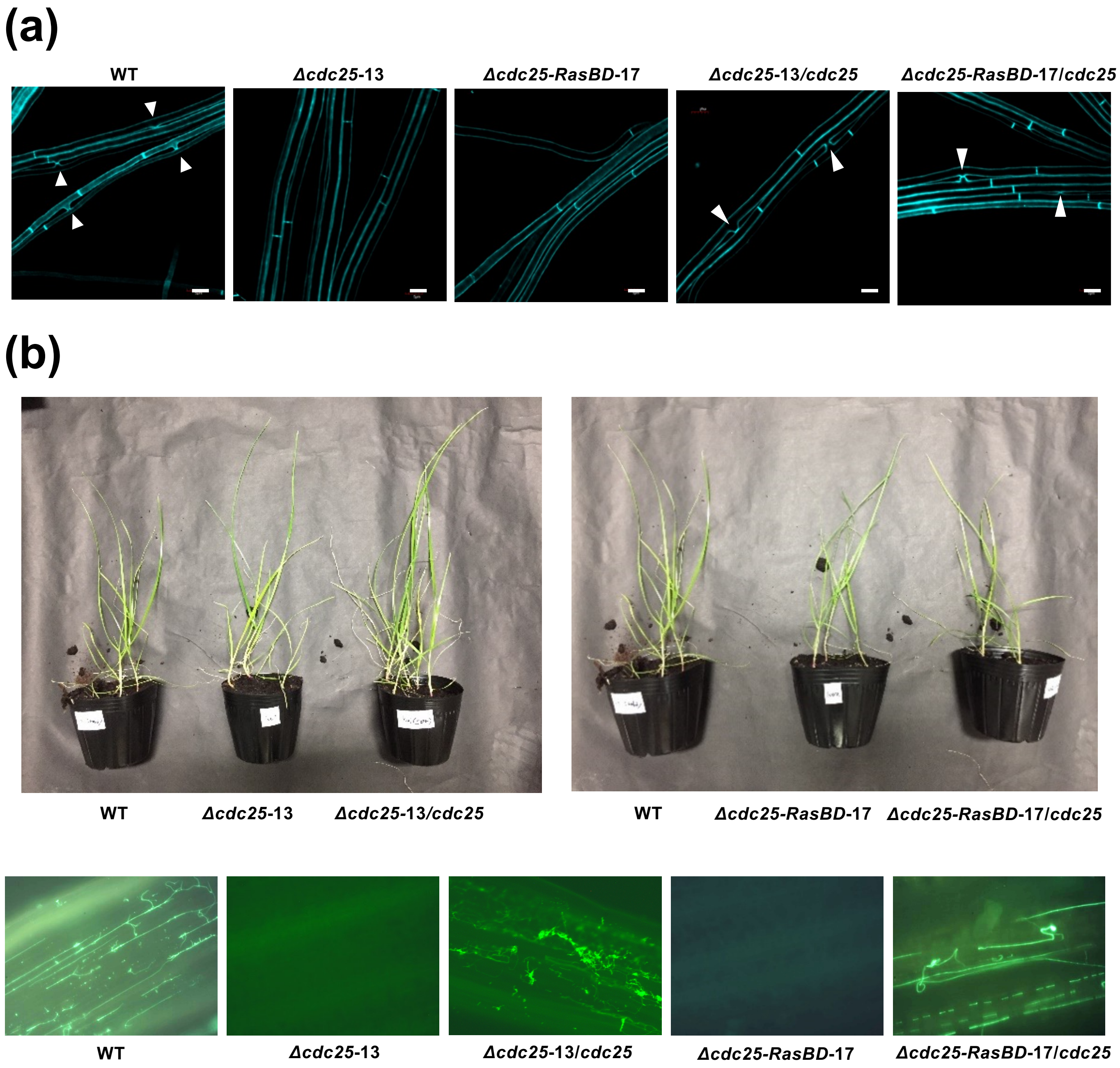

FIGURE S2 | (a) Hyphal fusion of *Epichloë festucae* wild type (WT), *cdc25* mutants, and complemented strains. Hyphal morphology and fusion were examined in *E. festucae* strains grown on water agar, stained with Calcofluor white, and observed using confocal laser scanning microscopy. Arrowheads indicate hyphal fusion events. Bars = 5 µm. **(b)** Infection of *E. festucae cdc25* mutants and complemented strains in perennial ryegrass. (top) Perennial ryegrass plants infected with *E. festucae* WT, *cdc25* mutants, and complemented strains. (bottom) Hyphae of *E. festucae* WT, *cdc25* mutants, and complemented strains within host tissues. Leaves inoculated with each strain were stained with aniline blue and observed under fluorescence microscopy.
