# Supplementary material for: Cdc25‐Mediated Activation of the Small GTPase RasB Is Essential for Hyphal Fusion and Symbiotic Infection of Epichloë festucae
Source: Mol Plant Pathol. 2026 Jan 28;27(1):e70210. doi: 10.1111/mpp.70210 (PMC12851848; doi:10.1111/mpp.70210)
Supplement: Supplementary file 3 — Figure S3: Alignment of the deduced amino acid sequence of EfRasB with RasB from fungal species. [file MPP-27-e70210-s010.pdf]

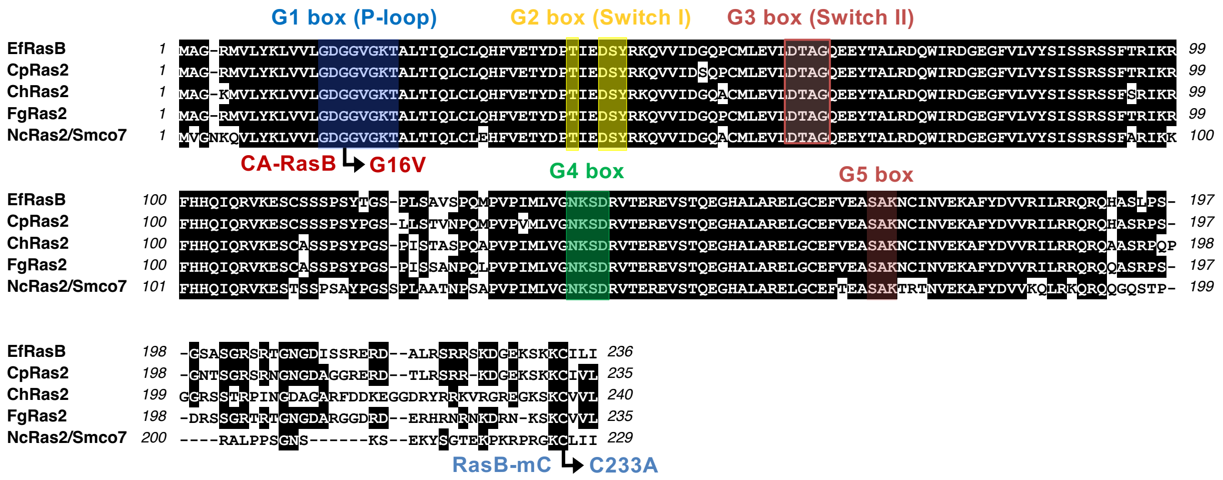

FIGURE S3 | Alignment of the deduced amino acid sequence of *Epichloë festucae* RasB (EfRasB) with RasB/Ras2 from *Claviceps purpurea* (CpRas2), *Colletotrichum higginsianum* (ChRas2), *Fusarium graminearum* (FgRas2), and *Neurospora crassa* (NcRas2/Smco7). Conserved domains among Ras GTPases are boxed. Amino acid substitutions introduced to generate the constitutively active (CA) form and the mutation at the geranylgeranylation site used for the yeast two-hybrid assay are indicated.
