# Supplementary material for: Cdc25‐Mediated Activation of the Small GTPase RasB Is Essential for Hyphal Fusion and Symbiotic Infection of Epichloë festucae
Source: Mol Plant Pathol. 2026 Jan 28;27(1):e70210. doi: 10.1111/mpp.70210 (PMC12851848; doi:10.1111/mpp.70210)
Supplement: Supplementary file 4 — Figure S4: Subcellular localization of GFP‐Cdc25 in hyphae of E. festucae. [file MPP-27-e70210-s007.pdf]

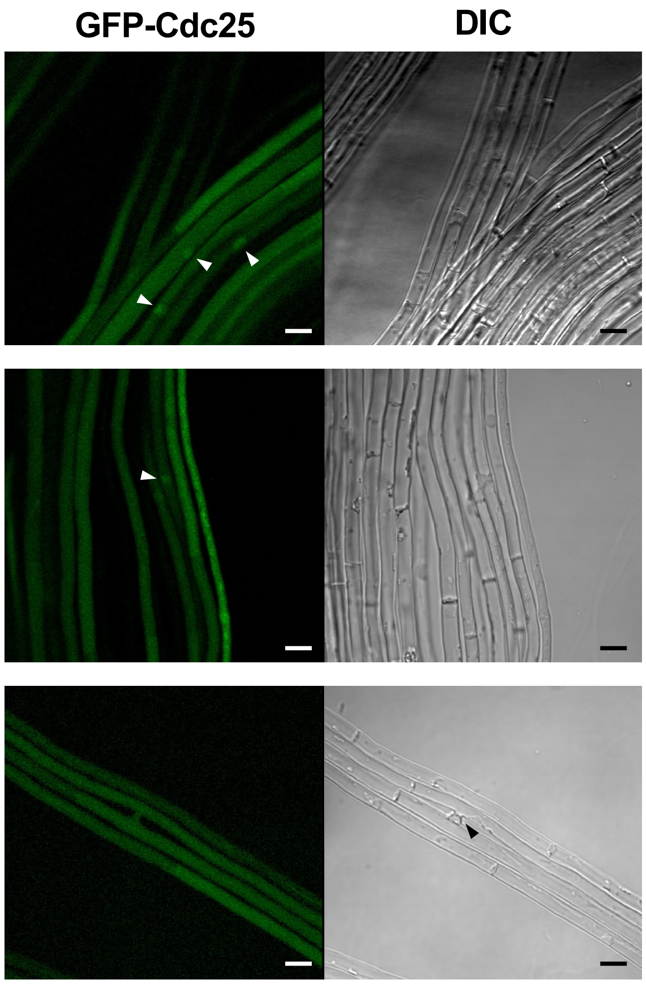

FIGURE S4 | Subcellular localization of GFP-Cdc25 in hyphae of *Epichloë festucae*. GFP-RasB was expressed under the control of the *TEF* promoter. Localization of GFP-Cdc25 was examined in three individual transformants. White and black arrowheads indicate the potential local accumulation of GFP-Cdc25 and the sites of hyphal fusion, respectively. Bars = 5 μm.
