# Supplementary material for: Cdc25‐Mediated Activation of the Small GTPase RasB Is Essential for Hyphal Fusion and Symbiotic Infection of Epichloë festucae
Source: Mol Plant Pathol. 2026 Jan 28;27(1):e70210. doi: 10.1111/mpp.70210 (PMC12851848; doi:10.1111/mpp.70210)
Supplement: Supplementary file 6 — Figure S6: Effect of overexpressing constitutively active RasB on hyphal morphology of E. festucae. [file MPP-27-e70210-s013.pdf]

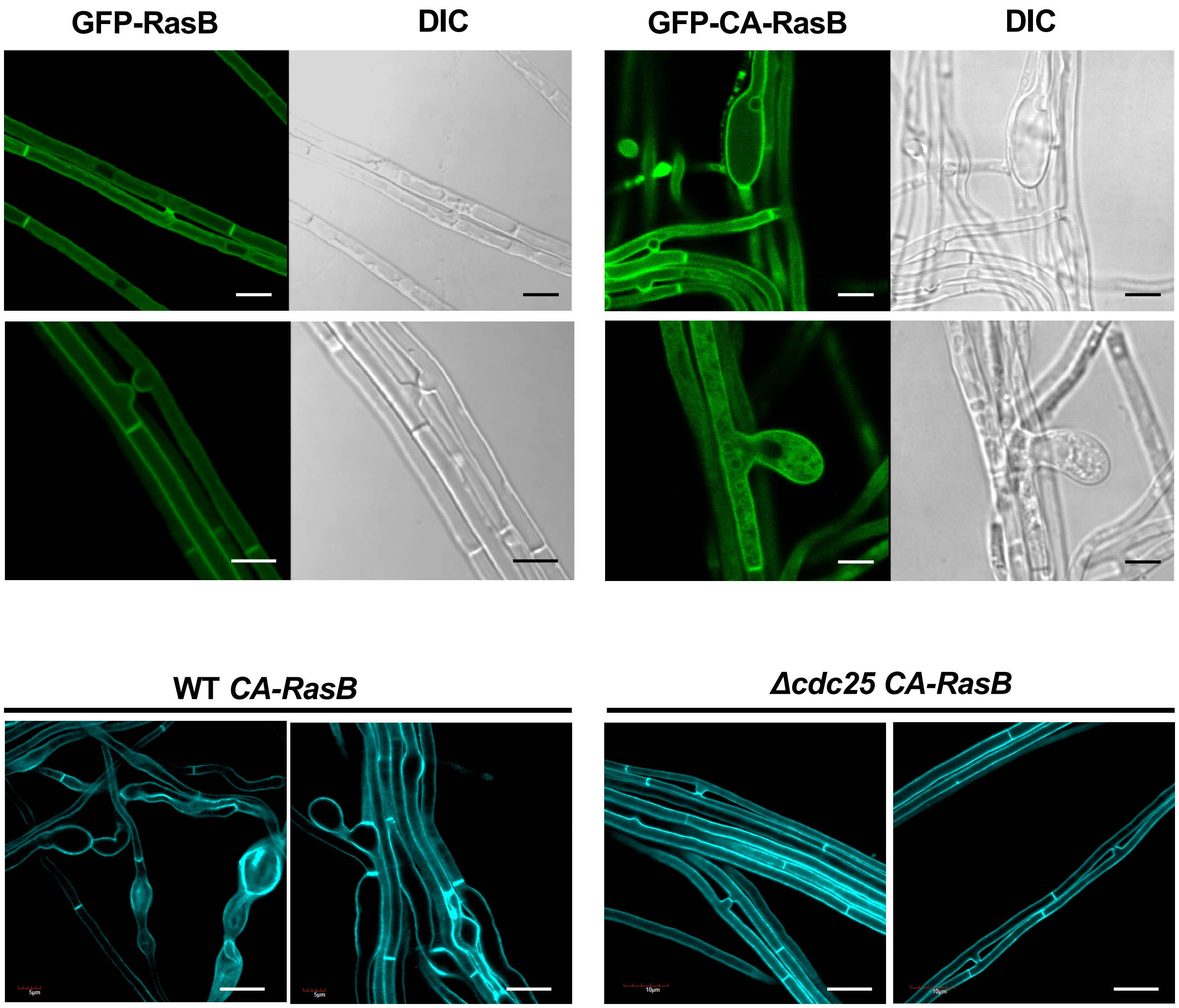

FIGURE S6 | Effect of overexpressing constitutively active RasB on hyphal morphology of *Epichloë festucae*.

(a) GFP-RasB or GFP-CA-RasB was expressed under the control of the *TEF* promoter. Localization of GFP fluorescence and hyphal morphology were observed in hyphae grown on water agar. Bars = 5 µm. **(b)** Hyphal morphology of *E. festucae* wild type (WT) or *Δcdc25* expressing CA-RasB under the control of *TEF* promoter. *E. festucae* transformants were grown on water agar, stained with Calcofluor white, and observed using confocal laser scanning microscopy. Bars = 10 µm.
