# Supplementary material for: Cdc25‐Mediated Activation of the Small GTPase RasB Is Essential for Hyphal Fusion and Symbiotic Infection of Epichloë festucae
Source: Mol Plant Pathol. 2026 Jan 28;27(1):e70210. doi: 10.1111/mpp.70210 (PMC12851848; doi:10.1111/mpp.70210)
Supplement: Supplementary file 7 — Figure S7: Strategy for deletion of E. festucae so gene. [file MPP-27-e70210-s012.pdf]

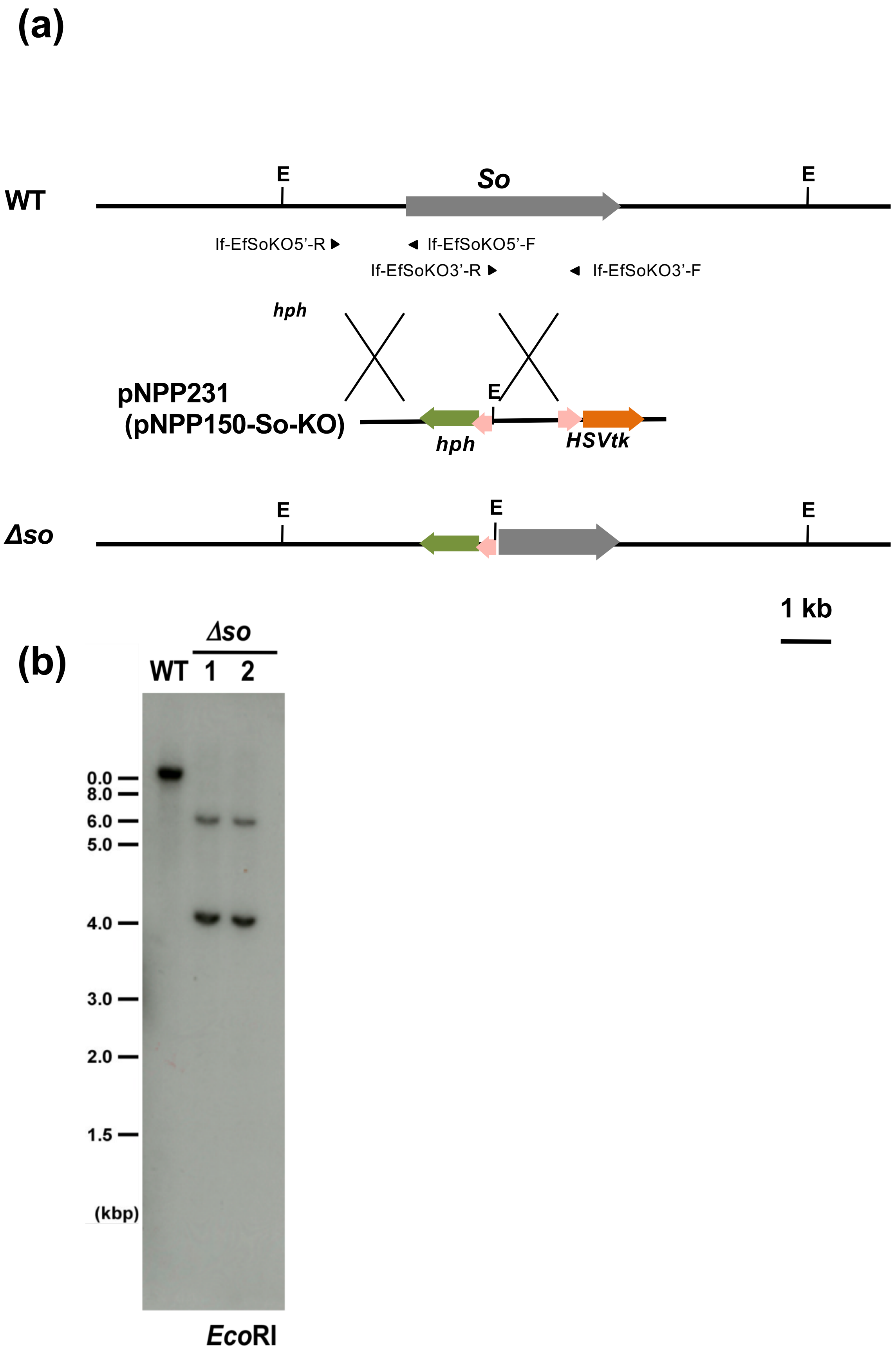

FIGURE S7 | Strategy for deletion of *Epichloë festucae so* gene.
(a) Physical map of the *so* wild-type genomic region and linear insert of *so* replacement construct pNPP231 (pNPP150-so-KO). E, *Eco*RI. **(b)** Autoradiograph of Southern blot of *Eco*RI-digested genomic DNA of *E. festucae* wild-type (WT) and *Δso* with [^{32}P]-labeled pNPP231. The sizes of marker DNA fragments are given in kb.
