# Supplementary material for: Cdc25‐Mediated Activation of the Small GTPase RasB Is Essential for Hyphal Fusion and Symbiotic Infection of Epichloë festucae
Source: Mol Plant Pathol. 2026 Jan 28;27(1):e70210. doi: 10.1111/mpp.70210 (PMC12851848; doi:10.1111/mpp.70210)
Supplement: Supplementary file 8 — Table S1: Summary of AlphaFold3‐predicted E. festucae Cdc25–RasB interfaces. [file MPP-27-e70210-s003.pdf]

**Table S1.** Summary of AlphaFold3-predicted *Epichloë festucae* Cdc25–RasB interfaces and domain architecture

| Model | Mean pLDDT | ipTM | pTM | Rank score | Mean PAE (Å) | Interface_residues (EfCdc25/RasB) | Interface_type | Major domains in EfCdc25 | Notes | Recycles |
|---|---|---|---|---|---|---|---|---|---|---|
| 0 | 85.1 | 0.77 | 0.51 | 0.84 | 7.6 | 890-1070 / 35-65 | RasGEF-Switch I/II | SH3, REM, RasGEF | Top-ranked model; stable interface | 10 |
| 1 | 85.0 | 0.76 | 0.51 | 0.83 | 7.8 | 895-1065 / 38-67 | RasGEF-Switch I/II | SH3, REM, RasGEF | Similar to model 0, minor RMSD shift | 10 |
| 2 | 85.2 | 0.76 | 0.51 | 0.83 | 7.5 | 880-1080 / 33-70 | RasGEF-Switch I/II | SH3, REM, RasGEF | Consistent binding orientation | 10 |
| 3 | 84.7 | 0.76 | 0.51 | 0.83 | 7.9 | 900-1075 / 30-68 | RasGEF-Switch I/II | SH3, REM, RasGEF | Slightly looser interface | 10 |
| 4 | 84.9 | 0.76 | 0.51 | 0.82 | 8 | 895-1070 / 34-66 | RasGEF-Switch I/II | SH3, REM, RasGEF | Lower rank; similar topology | 10 |

pLDDT, predicted Local Distance Difference Test; ipTM, interface predicted Template Modelling; pTM, predicted Template Modelling; PAE, Predicted Aligned Error; RMSD, Root Mean Square Deviation.
