# Supplementary material for: Cdc25‐Mediated Activation of the Small GTPase RasB Is Essential for Hyphal Fusion and Symbiotic Infection of Epichloë festucae
Source: Mol Plant Pathol. 2026 Jan 28;27(1):e70210. doi: 10.1111/mpp.70210 (PMC12851848; doi:10.1111/mpp.70210)
Supplement: Supplementary file 9 — Table S2: mpp70210‐sup‐0009‐TableS2.pdf. Epichloë festucae strains used in this study. [file MPP-27-e70210-s008.pdf]

Table S2. *Epichloë festucae* strains used in this study.

Fungal strains	Relevant characteristics	Reference
Epichloë festucae		
Fl1	Wild type	Young *et al.* 2005[*1]
RPA41	Fl1/pNPP1; Hyg^R	This study
RPA112	Fl1/pNPP1; Hyg^R	This study
RPA519	Fl1/pNPP1; Hyg^R	This study
Δham8-1	Fl1/*Δham8*::P*trpC*-*hph*; Hyg^R	Tanaka *et al.* 2020[*2]
ΔmpkB-1	Fl1/*ΔmpkB*::P*trpC*-*hph*; Hyg^R	Tanaka et al. 2020[*2]
Δso	Fl1/*Δso*::P*trpC*-*hph* (pNPP231); Hyg^R	This study
ΔnoxA	Fl1/*ΔnoxA*::P*trpC*-*hph*; Hyg^R	Tanaka *et al.* 2006[*3]
Δcdc25-13	Fl1/*Δcdc25*::P*trpC*-*hph* (pNPP233); Hyg^R	This study
Δcdc25-RasBD-16	Fl1/*Δcdc25-RasBD*::P*trpC*-*hph* (pNPP234); Hyg^R	This study
Δcdc25-RasBD-17	Fl1/*Δcdc25-RasBD*::P*trpC*-*hph* (pNPP234); Hyg^R	This study
Δcdc25-RasBD-18	Fl1/*Δcdc25-RasBD*::P*trpC*-*hph* (pNPP234); Hyg^R	This study
Δcdc25-RasBD-25	Fl1/*Δcdc25-RasBD*::PtrpC-hph (pNPP234); Hyg^R	This study
Δcdc25-RasBD-26	Fl1/*Δcdc25-RasBD*::P*trpC*-*hph* (pNPP234); Hyg^R	This study
Δcdc25-13-GFP	*Δcdc25-13*/pNPP99; Hyg^R, Gen^R	This study
Δcdc25-RasBD-17-GFP	*Δcdc25-RasBD-17*/pNPP99; Hyg^R, Gen^R	This study
Δcdc25-13/cdc25	*Δcdc25-13*/pNPP241; Hyg^R, Gen^R	This study
Δcdc25-RasBD-17/cdc25	*Δcdc25-RasBD-17*/pNPP241; Hyg^R, Gen^R	This study
GFP-RasB	Fl1/pNPP223; Hyg^R	This study
GFP-CA-RasB	Fl1/pNPP224; Hyg^R	This study
GFP-Cdc25	Fl1/pNPP222; Hyg^R	This study
CA-RasB	Fl1/pNPP225; Hyg^R	This study
Δcdc25-13/CA-RasB	*Δcdc25-13*/pNPP225, pSF17; Hyg^R, Gen^R	This study
Δcdc25-RasBD-17/CA-RasB	*Δcdc25-RasBD-17*/pNPP225, pSF17; Hyg^R, Gen^R	This study
ΔmpkB/CA-RasB	*ΔmpkB*/pNPP225, pSF17; Hyg^R, Gen^R	This study
ΔnoxA/CA-RasB	*ΔnoxA*/pNPP225, pSF17; Hyg^R, Gen^R	This study

[*1] Young et al. (2005) Mol Genet Genomics 274: 13–29. https://doi.org/10.1007/s00438-005-1130-0

[*2] Tanaka et al. (2020) Mol Microbiol 114: 626–640. https://doi.org/10.1111/mmi.14568

[*3] Tanaka et al. (2006) Plant Cell 18: 1052–1066. https://doi.org/10.1105/tpc.105.039263
