# Supplementary material for: Cdc25‐Mediated Activation of the Small GTPase RasB Is Essential for Hyphal Fusion and Symbiotic Infection of Epichloë festucae
Source: Mol Plant Pathol. 2026 Jan 28;27(1):e70210. doi: 10.1111/mpp.70210 (PMC12851848; doi:10.1111/mpp.70210)
Supplement: Supplementary file 10 — Table S3: Vectors for Epichloë festucae transformation used in this study. [file MPP-27-e70210-s009.pdf]

Table S3. Plasmids for *Epichloë festucae* transformation used in this study

Vector name	Base vector	Insert	Primers used to amplify insert	References	Description
**Base vectors**					
pNPP1	-	-	-	Kayano et al. 2013	Vector used for REMI, Amp^R/Hyg^R,
pPN94	-	-	-	Takemoto et al. 2006	Base vector for gene expression under TEF promoter, Amp^R/Hyg^R
pSF17.1	-	-	-	Tanaka et al. 2008	Base vector for complementation and co-transformation, Amp^R/Gen^R
pNPP141 (pPN94-GFP-3GA)	-	-	-	Kayano et al. 2013	Vector for expression of GFP-tagged protein, Amp^R/Hyg^R
pNPP150	-	-	-	Niones and Takemoto 2015	Base vector for KO (HSVtk), Amp^R/Hyg^R
pNPP99	-	-	-	Kayano et al. 2013	Expression of GFP, Amp^R/Gen^R
**Plasmids for gene knock out and complementation**					
pNPP231 (pNPP150-So-KO)	pNPP150	5'So-PtrpC-hph-3'So	IF So-KO5-F, IF So-KO5-R, IF So-KO3-F, IF So-KO3-R	This study	So KO, Amp^R/Hyg^R
pNPP232 (pNPP150-RPA112-KO)	pNPP150	5'C2857_000796-PtrpC-hph-3'C2857_000796	IF RPA112-KO5-F, IF RPA112-KO5-R, IF RPA112-KO3-F, IF RPA112-KO3-R	This study	C2857_000796 KO, Amp^R/Hyg^R
pNPP233 (pNPP150-Cdc25-KO)	pNPP150	5'Cdc25-PtrpC-hph-3'Cdc25	IF Cdc25-KO5-F1, IF Cdc25-KO5-R1, IF Cdc25-KO3-F, IF Cdc25-KO3-R	This study	Cdc25 KO, Amp^R/Hyg^R
pNPP234 (pNPP150-Cdc25-RasBD-KO)	pNPP150	5'Cdc25RasBD-PtrpC-hph-3'Cdc25	IF Cdc25-KO5-F2, IF Cdc25-KO5-R2, IF Cdc25-KO3-F, IF Cdc25-KO3-R	This study	Cdc25 Ras binding domain KO, Amp^R/Hyg^R
pNPP235 (pNPP150-RasB-KO1)	pNPP150	5'RasB-PtrpC-hph-3'RasB	IF RasB-KO5-F, IF RasB-KO5-R, IF RasB-KO3-F1, IF RasB-KO3-R1	This study	RasB KO, Amp^R/Hyg^R
pNPP236 (pNPP150-RasB-KO2)	pNPP150	5'RasB-PtrpC-hph-3'RasB2	IF RasB-KO5-F, IF RasB-KO5-R, IF RasB-KO3-F2, IF RasB-KO3-R2	This study	RasB KO, Amp^R/Hyg^R
pNPP241 (pSF17-Cdc25)	pSF17.1	*Cdc25*	pSF17-cdc25-F2, pSF17-cdc25-R2	This study	Complementation of *cdc25* , Amp^R/Gen^R
**Plasmids for gene expression in *E. festucae***					
pNPP222 (pPN94-GFP-Cdc25)	pNPP141	GFP-Cdc25	IF pPN94-GFP-3GA-Cdc25-F, IF pPN94-GFP-3GA-Cdc25-R	This study	Expression of GFP-Cdc25, Amp^R/Hyg^R
pNPP223 (pPN94-GFP-RasB)	pNPP141	GFP-RasB	IF pPN94-GFP-3GA-RasB-F, IF pPN94-GFP-3GA-RasB-R	This study	Expression of GFP-RasB, Amp^R/Hyg^R
pNPP224 (pPN94-GFP-CA-RasB)	pNPP141	GFP-CA-RasB	IF pPN94-GFP-3GA-RasB-F, IF pPN94-GFP-3GA-RasB-R	This study	Expression of GFP-CA-RasB, Amp^R/Hyg^R
pNPP225 (pPN94-CA-RasB)	pPN94	CA-RasB	IF pPN94-RasB-F, IF pPN94-RasB-R, IF RasB-DA-F, IF RasB-DA-F2, IF RasB-DA-F3, IF RasB-DA-R	This study	Expression of CA-RasB, Amp^R/Hyg^R
