# Supplementary material for: Cdc25‐Mediated Activation of the Small GTPase RasB Is Essential for Hyphal Fusion and Symbiotic Infection of Epichloë festucae
Source: Mol Plant Pathol. 2026 Jan 28;27(1):e70210. doi: 10.1111/mpp.70210 (PMC12851848; doi:10.1111/mpp.70210)
Supplement: Supplementary file 11 — Table S4: Vectors for yeast transformation used in this study. [file MPP-27-e70210-s011.pdf]

Table S4. Plasmids for yeast two-hybrid assay used in this study.

Plasmid name	Base vector	Insert	Primers used to amplify insert	References	Description
**Base vectors**					
pGADT7	-	-	-	Clontech	Amp^R/LEU2
pGBKT7	-	-	-	Clontech	Kan^R/TRP1
**Plasmids for yeast two-hybrid assay**					
pNPP216 (pGADT7 Cdc25)	pGADT7	Cdc25	IF-pGADT7-Cdc25-F, IF-pGADT7-Cdc25-R	This study	Full length Cdc25
pNPP217 (pGBKT7 RasA-mC)	pGBKT7	RasA-mC	IF-pGBKT7-RasA-F, IF-pGBKT7-RasAmC-R	This study	Full length RasA with mutation in CaaX motif (C213A)
pNPP218 (pGBKT7 RasB-mC)	pGBKT7	RasB-mC	IF-pGBKT7-RasB-F, IF-pGBKT7-RasBmC-R	This study	Full length RasB with mutation in CaaX motif (C233A)
pNPP219 (pGBKT7 RasC-mC)	pGBKT7	RasC-mC	IF-pGBKT7-RasC-F, IF-pGBKT7-RasCmC-R	This study	Full length RasC with mutation in CaaX motif (C267A)
pNPP220 (pGBKT7 RhbA-mC)	pGBKT7	RhbA-mC	IF-pGBKT7-RhbA-F, IF-pGBKT7-RhbAmC-R	This study	Full length RhbA with mutation in CaaX motif (C183A)
pNPP221 (pGBKT7 KrevA-mC)	pGBKT7	KrevA-mC	IF-pGBKT7-KrevA-F, IF-pGBKT7-KrevAmC-R	This study	Full length KrevA with mutation in CaaX motif (C216A)
