# Supplementary material for: Cdc25‐Mediated Activation of the Small GTPase RasB Is Essential for Hyphal Fusion and Symbiotic Infection of Epichloë festucae
Source: Mol Plant Pathol. 2026 Jan 28;27(1):e70210. doi: 10.1111/mpp.70210 (PMC12851848; doi:10.1111/mpp.70210)
Supplement: Supplementary file 12 — Table S5: Primers for vector construction used in this study. [file MPP-27-e70210-s006.pdf]

Table S5. Primers used in this study

Primer name	Sequence
cdc25-F	TGCGAAACGAGCAAAGACAG
cdc25-R	TATGCCGCATTGCTACGTAC
Ppro41-F	CTGAGTTGGACAAATATGGC
Ppro41-R	GCCACTTCGGCCTATTCTCC
IF So-KO5-F	TTAGGTGACACTATACATGTCAAGTCAGCG
IF So-KO5-R	CAGCTGCTCGAGTTCCCCCCTCTGTTTCTC
IF So-KO3-F	TGAGTCGTATTAATTATGGAAACCAGCGTC
IF So-KO3-R	AACCTGGCTTATCGATGAGAATGCTTCCCA
IF Cdc25-KO5-F1	ATGCCTGCAGGTCGAATAAGTTGGTGAGACTTCCC
IF Cdc25-KO5-R1	ATCCTCTAGAGTCGAGTTTGTGCCGAAGCGCACAA
IF Cdc25-KO5-F2	ATGCCTGCA+C8GGTCGAGCAACTGCTCTCCGAGCAAG
IF Cdc25-KO5-R2	ATCCTCTAGAGTCGAGTAGTTGTTGAGAGTTCGGC
IF Cdc25-KO3-F	TACCGAGCTCGAATTATCGAACCATCGGGGTCAAC
IF Cdc25-KO3-R	TATCATCGATGAATTTTAGATTCCATGTCGTCAGG
IF RPA112-KO5-F	ATGCCTGCAGGTCGACTGTGATATCAACGTCCCTG
IF RPA112-KO5-R	ATCCTCTAGAGTCGATTCATTTCTCGAAGCCGTCG
IF RPA112-KO3-F	TACCGAGCTCGAATTGAAAGACAGTCCTCGTCTTG
IF RPA112-KO3-R	TATCATCGATGAATTATCGGCCTTGACCAGTTCCT
IF-pGADT7-Cdc25-F	GGAGGCCAGTGAATTCATGGAGGGCATTGTGGGAA
IF-pGADT7-Cdc25-R	TCATCTGCAGCTCGAGTCATAGAAACCCGGACTCG
IF-pGBKT7-RasA-F	CATGGAGGCCGAATTCATGGCCGCATCCACAAAGT
IF-pGBKT7-RasAmC-R	GCAGGTCGACGGATCCTCACATAATGATAGCCTTG
IF-pGBKT7-RasB-F	CATGGAGGCCGAATTCATGGCGGGCCGTATGGTGT
IF-pGBKT7-RasBmC-R	GCAGGTCGACGGATCCTCATATAAGGATGGCTTTT
IF-pGBKT7-RasC-F	CATGGAGGCCGAATTCATGTCTTCTCAGCTGGAGC
IF-pGBKT7-RasCmC-R	GCAGGTCGACGGATCCTTACCAGGCCCGCAGCTTC
IF-pGBKT7-RhbA-F	CATGGAGGCCGAATTCATGCCTGCGCCAAAGCAGA
IF-pGBKT7-RhbAmC-R	GCAGGTCGACGGATCCCTACATGAGAGAGGCGTTG
IF-pGBKT7-KrevA-F	CATGGAGGCCGAATTCATGGCGCCTCGATTCCACG
IF-pGBKT7-KrevAmC-R	GCAGGTCGACGGATCCCTACAAGATTACGGCTCTG
IF pSF17 cdc25-F2	GAATTCATCGATGATGCAGTTAAACTCGTTCTCGT
IF pSF17 cdc25-R2	ACCGGCAGATCTGATGGGGCTGTTTTGATGAAAAG
IF RasB-KO5-F	ATGCCTGCAGGTCGACCAAGGGTGAGGTTGTGTAT
IF RasB-KO5-R	ATCCTCTAGAGTCGATGGAACCTTGCGTGCCGAAA
IF RasB-KO3-F1	TACCGAGCTCGAATTCGGCAACAACGAAGCGAGTG
IF RasB-KO3-R1	TATCATCGATGAATTAGGGCTGGCTGTCTGTAAGT
IF RasB-KO3-F2	TACCGAGCTCGAATTACGGGGTCACCTCTTTCCGC
IF RasB-KO3-R2	TATCATCGATGAATTTCATCTCTTCATGTCGACCT
IF pPN94-RasB-F	AACCTCTAGAGGATCATGGCGGGCCGTATGGTGTT
IF pPN94-RasB-R	ACGTTAAGTGCGGCCTCATATAAGGATGCATTTTT
IF RasB-DA-F	GACGTCGGCGTAGGAAAGAC
IF RasB-DA-R	TCCTACGCCGACGTCTCCCA
IF RasB-DA-F2	ACAAGCTTGTGGTTCTGGGAGACGTCGGCGTAGGA
IF RasB-DA-F3	GGCGGGCCGTATGGTGTTGTACAAGCTTGTGGTTC
IF pPN94-GFP-3GA-RasB-F	TGCTGGTGCTGAATTCATGGCGGGCCGTATGGTGT
IF pPN94-GFP-3GA-RasB-R	ACGTTAAGTGCGGCCTCATATAAGGATGCATTTTT
IF pPN94-GFP-3GA-Cdc25-F	TGCTGGTGCTGAATTCATGGAGGGCATTGTGGGAA
IF pPN94-GFP-3GA-Cdc25-R	ACGTTAAGTGCGGCCTCATAGAAACCCGGACTCGG

Extension sequences for In-fusion reaction are in red letters.

Mismatches to introduce amino acid substitution are highlighted in blue letters.

Extension sequence for in-fusion reaction are shown in red letters.
