# Supplementary material for: Cdc25‐Mediated Activation of the Small GTPase RasB Is Essential for Hyphal Fusion and Symbiotic Infection of Epichloë festucae
Source: Mol Plant Pathol. 2026 Jan 28;27(1):e70210. doi: 10.1111/mpp.70210 (PMC12851848; doi:10.1111/mpp.70210)
Supplement: Supplementary file 13 — Table S6: Yeast strains used in this study. [file MPP-27-e70210-s005.pdf]

Table S6. Yeast strains used in this study

Yeast strains	Relevent characteristics	References
Saccharomyces cerevisiae		
AH109	MATa, trp1-901, Leu2-3, 112, ura3-52, his3-200, gal4⊿, gal80⊿, LYS2::GAL1UAS-GAL1TATA-HIS3, MEL1, GAL2UAS-GAL2TATA-ADE2, URA::MELIUAS-MEL1TATA-laeZ	Clontech
AH109 (Cdc25/empty)	AH109/pNPP216; pGBKT7; *LEU/TPR1*	This study
AH109 (empty/RasA)	AH109/pGADT7; pNPP217; LEU/TPR1	This study
AH109 (empty/RasB)	AH109/pGADT7; pNPP218; LEU/TPR1	This study
AH109 (empty/RasC)	AH109/pGADT7; pNPP219; LEU/TPR1	This study
AH109 (empty/RhbA)	AH109/pGADT7; pNPP220; LEU/TPR1	This study
AH109 (empty/KrevA)	AH109/pGADT7; pNPP221; LEU/TPR1	This study
AH109 (Cdc25/RasA)	AH109/pNPP216; pNPP217; *LEU/TPR1*	This study
AH109 (Cdc25/RasB)	AH109/pNPP216; pNPP218; *LEU/TPR1*	This study
AH109 (Cdc25/RasC)	AH109/pNPP216; pNPP219; *LEU/TPR1*	This study
AH109 (Cdc25/RhbA)	AH109/pNPP216; pNPP220; *LEU/TPR1*	This study
AH109 (Cdc25/KrevA)	AH109/pNPP216; pNPP221; *LEU/TPR1*	This study
