# Supplementary material for: Cdc25‐Mediated Activation of the Small GTPase RasB Is Essential for Hyphal Fusion and Symbiotic Infection of Epichloë festucae
Source: Mol Plant Pathol. 2026 Jan 28;27(1):e70210. doi: 10.1111/mpp.70210 (PMC12851848; doi:10.1111/mpp.70210)
Supplement: Supplementary file 5 — Figure S5: Attempted disruption of rasB gene in E. festucae. [file MPP-27-e70210-s004.pdf]

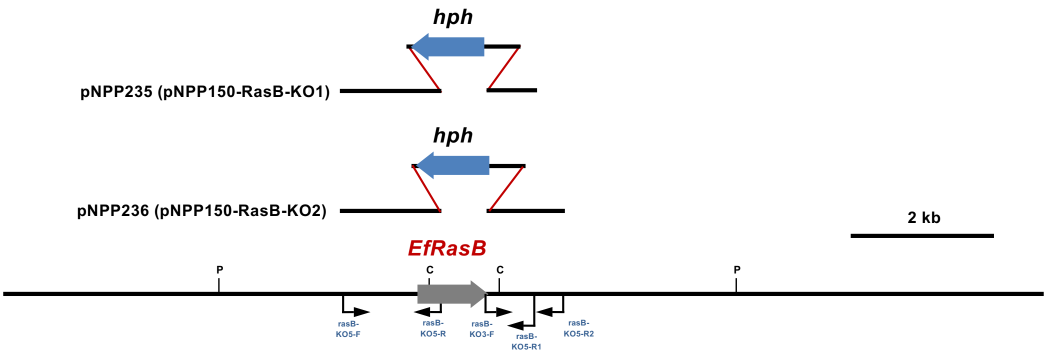

FIGURE S5 | Attempted disruption of *rasB* gene in *Epichloë festucae*.
Physical map of the *rasB* wild-type genomic region and linear inserts of *rasB* replacement constructs pNPP150-RasB-KO1 and pNPP150-RasB-KO2. P, *Pst*I.
